# The effect of motivational interviewing on oral healthcare knowledge, attitudes and behaviour of parents and caregivers of preschool children: an exploratory cluster randomised controlled study

**DOI:** 10.1186/s12903-015-0068-9

**Published:** 2015-09-02

**Authors:** Rahul Naidu, June Nunn, Jennifer D. Irwin

**Affiliations:** School of Dentistry, The University of the West Indies, St Augustine, Trinidad and Tobago; School of Dental Science, Trinity College Dublin, Dublin 2, Ireland; School of Health Studies, Western University, Ontario, Canada

**Keywords:** Preschool children, Oral health motivational interviewing, West Indies

## Abstract

**Background:**

Motivational Interviewing (MI) has been used across primary healthcare and been shown to be effective in reducing the prevalence of early childhood caries (ECC) in preschool children. This study aimed to compare the effect of MI, in contrast to traditional dental health education (DHE), on oral health knowledge, attitudes, beliefs and behaviours among parents and caregivers of preschool children in Trinidad.

**Method:**

The design of this exploratory study included a cluster randomised controlled trial and semi-structured focus groups. Six preschools (79 parents and caregivers) in Eastern Trinidad were randomly assigned to a test or control group (3 preschools in each group). Parents and caregivers in the test-group (*n* = 25) received a talk on dental health using an MI approach and the control-group (*n* = 54) received a talk using traditional DHE. Both groups received additional, written dental health information. The MI group also received two telephone call follow-ups as part of the MI protocol. Both groups were given questionnaires before the talks and four months later. Question items included oral health knowledge, beliefs, attitudes, brushing behaviour, oral health self-efficacy, oral health fatalism and a specific instrument to asses ‘readiness for change’, the Readiness Assessment of Parents Concerning Infant Dental Decay (RAPIDD). Participants in the test-group were also invited to take part in a focus group to share their views on the dental health talk.

**Results:**

At four month follow-up, knowledge items on fluoride use, tooth brushing, dietary practice and dental attendance increased in both the test (DHE + MI) and control (DHE) groups ((*p* < 0.05, Chi Square test). In the test-group there were increases in mean child tooth brushing frequency and reduction in oral health fatalism (*p* < 0.05 *t*-test). Findings from a thematic analysis of the focus group suggested that the MI talk and telephone follow-up were well accepted and helpful in supporting parent and caregiver efforts to improve oral health practices for their preschool children.

**Conclusion:**

In this exploratory controlled study there was some evidence that using an MI approach when delivering oral health information had a positive effect on parent/ caregiver oral health knowledge, attitudes and behaviours compared to traditional DHE. There is need for further research involving the use of brief-counselling techniques in this Caribbean population.

## Background

Parents and caregivers can be considered gatekeepers for the oral healthcare of preschool children, therefore their oral health knowledge, beliefs, attitudes and behaviour may directly, or indirectly, influence early childhood oral health.

**Fig. 1:**
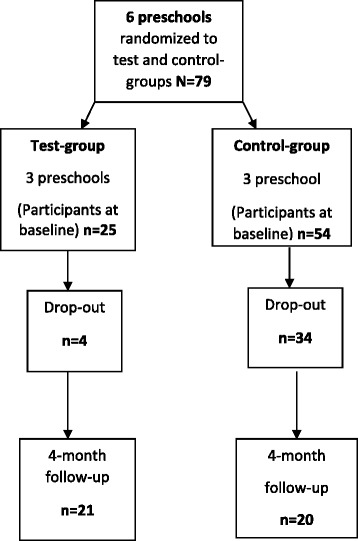
Response to the study

Traditional dental health education approaches with a main focus on improving parental oral health knowledge have not been effective in improving preschool children’s oral health [1–3]. For instance, traditional generic recommendations such as ‘brush your child’s teeth twice a day and reduce consumption of sweet snacks and drinks’ have had limited success in changing oral health practices [4].

Behaviour change techniques (BCT), including face-to-face counselling, have been effective across primary healthcare [5]. BCTs have been described as an ‘observable, replicable and irreducible component of an intervention designed to alter or redirect causal processed that regulate behaviour’ [6]. One such BCT is Motivational Interviewing (MI), defined as “*a person-centred counselling style for addressing the common problem of ambivalence about change*” [7]. MI differs radically from traditional health education approaches in which it is assumed from the outset that a person is prepared to act upon information imparted to them by an expert (health professional/ educator). Rather than the health professional assuming the role of ‘expert’, MI places the client/ patient in that role, letting them decide how to interpret and integrate information in the context of their lives own and social circumstances and whether it is relevant for them [3].

During MI the health professional/educator attempts to resolve ambivalence toward change in the client/patient and ‘evoke’ motivation that is already there by the eliciting ‘change-talk’ and using ‘reflective listening’. This means asking questions like: *What do you want to change? How might you go about it? What are the best reasons for doing it and how important is it you?* [7]. MI therefore sees behaviour change as a partnership between the client / patient and counsellor / health educator that respects autonomy, enabling the client / patient to feel engaged, understood and empowered.

Though not strictly a theory of behaviour change, MI does share some elements of the Transtheoretical Model (TTM) as proposed by Prochaska et al. [8]. Through work with people with addictive behaviour, the TTM was developed to understand self-initiated and professionally assisted changes in health behaviour. The TTM presents a series of key stages though which an individual must pass before adopting a new behaviour. These stages are (a) *Precontemplative,* (individual has no awareness of the problem or intention to change behaviour) (b) *Contemplative* (Individual aware of problem and thinking about change) (c) *Preparation* (Individual has intention to change but not yet ready)*,* (d) *Action* (Individual attempts behaviour change*),* and (e) *Maintenance* (individual consolidates change to prevent relapse) [9]. As in MI, the concept of increasing self-efficacy and personal empowerment are believed to be key elements in the process of change, these also being fundamental aims of health promotion.

Despite a sizable body of evidence from medical research, the potential of MI in dental health care is less well understood [10]. A recent systematic review on the effectiveness of MI compared to conventional health education (CE) suggested that MI outperformed CE in improving oral health behaviours in infants and preschool children, mainly in relation to oral hygiene, but not in dietary habits [10]. This evidence is largely based on studies by Weinstein et al. and Harrison et al. [11–13]. These studies reported findings of a two-year randomized controlled trial of MI involving 240 infants aged 6–18 months from Punjabi Sikh immigrant families living in British Columbia, Canada. In this blinded, randomized control trial, the experimental group received a dental health pamphlet and video, along with MI delivered by trained Indo-Canadian women lay counsellors (who were not health professionals), using a specifically developed protocol. Mothers in the control group received only the initial pamphlet and video and no telephone follow-up. After two years, a 46 % lower prevalence of decayed tooth surfaces was reported in children whose mother had received MI compared to those whose mothers received only pamphlets and videos. It should be stated that in these studies the oral health behaviour that most likely related to the reduction in ECC, was that families in the MI group attended more routinely for fluoride varnish application than the control families. This could also suggest that mothers in the MI group felt more positive about oral health care for their children and motivated to access preventive dental services.

Less promising results were reported in a randomized study of over 1000 low-income African-American families with children (0–5 years) [14]. The test-group involved MI for caregivers, a 40-min discussion about what changes they could make to achieve oral health goals for their child delivered by trained interviewers and a 15-min health education DVD. The control-group received the health educational DVD only. At two-year follow-up, apart from ‘checking the child’s mouth for pre-cavities’, there were no statistically significant differences in oral health behaviour of caregivers between test and control groups and no statistically significant difference in the development of untreated caries in the children.

The potential of MI in improving oral health care may therefore still be considered controversial [10]. Nevertheless, a recent evidence-based national clinical guidelines for caries prevention from the UK states “oral health promotion interventions should be based on recognised health theory behaviour and models such as MI [15].

Previous research as shown the prevalence and severity of ECC in Trinidad (an island nation in the West Indies) to be related to parental oral health behaviours and access to dental care [16] as well as families with preschool children being in need of practical support to overcome barriers to achieving effective preventive care [17]. However, no research has been conducted in the English-speaking Caribbean, assessing the effectiveness and appropriateness of community-based oral health promotion that includes the use of brief-counselling approaches such as MI for families with preschool children.

The aim of this study was to assess the effect of an MI approach delivered in a group-setting, on parent /caregiver oral health knowledge, beliefs, attitudes and behaviour toward the oral healthcare of their preschool children, by contrast with traditional dental health education (DHE), in an exploratory community-based study.

### Objectives

- To assess the effect of MI on participants’ oral health knowledge, beliefs and attitudes, tooth brushing behaviour, oral health self-efficacy and oral health fatalism compared to DHE.
- To assess the effect of MI on participants’ ‘readiness for change’, compared to DHE.
- To assess the effectiveness of MI delivered in a group-setting.
- To explore participants’ views and opinions of the MI intervention.

## Method

To achieve the objectives of this exploratory study two study designs were employed, a cluster randomised controlled trial and semi-structured focus groups. Quantitative methods were used to test the effectiveness of MI compared to DHE and a qualitative approach employed to explore participant experience with the MI intervention.

### Sample selection

The accessible population group for the MI intervention were families of children attending preschools within the catchment area of the Arima District Health Facility (Eastern Trinidad). This Health Centre also housed the local dental clinic, staffed by Dental Nurses (the equivalent of dental therapists in the UK), who would be assisting in the research.

The sampling frame consisted of 12 preschools on a contact list for community dental outreach activity by Dental Nurses working at the Arima District Health Facility. To enable involvement of the two Dental Nurses in this research concurrent to their clinical commitments in the District Health Facility, it was decided that a maximum of six preschool would be manageable within the time-frame of this pilot study. The 6 preschools (clusters) were drawn randomly from the contact list and subsequently assigned to the test group (DHE + MI) or the control group (DHE), with 3 preschool clusters in each group. The placement of preschools in a particular study group was based on simple randomization (preschools numbered 1–6 and these numbers randomly assigned to test or control group).

### Approval and consent

Ethical approval was obtained from the Faculty of Medical Sciences Research Ethics Committee (The University of the West Indies). For the selected preschools, letters of request to be included in the study were sent to the head teacher / preschool administrator, for each preschool. When approval was obtained, packages were then hand–delivered by a dental nurse to each preschool. These packages contained information about the study and a consent form requesting parent/caregiver participation and the study questionnaire. These documents were distributed to parents/caregivers via the head teachers, who were also asked to collate the returned consent forms and completed questionnaires, for collection by the study team.

### Experimental design and MI protocol

- Control group *– (Dental Health Education)*
  All participants (parents and caregivers) in the control group were given a 30 -min talk (as a group) on dental care of preschool children’s teeth by a Dental Nurse. This talk included advice on diet, oral hygiene, fluoride use and dental attendance. At the end of the talk, participants were given a DHE leaflet reinforcing the information to take home. All participants in the control-group were given dental health products (toothpaste samples and floss) as a token of appreciation for taking part in the study. The three control group-talks included 6, 13 and 35 participants, respectively.
- Test group *– (Motivational Interviewing & Dental Health Education)*
  Participants in the intervention group received a 30-min talk (as a group) based on a Motivational Interviewing approach, delivered by an MI counsellor /educator (RN) (a dentist trained in MI), assisted by a Dental Nurse. Training of the MI counsellor /educator involved a one-day course (8 h) on Motivational Interviewing and coaching skills for health professionals that included both applied (hands-on) and theoretical elements, with the theoretical materials also made available for self-study.
  The group-talk was based on an MI protocol designed to aid delivery of oral health information to families with young children: *Motivate your Dental Patient: A workbook -Public Health / Paediatric Edition* [18].
  All participants in the test group received the same DHE information leaflet as the control group along with toothpaste samples as tokens of appreciation. All the talks took place at the preschools after a normal school day. The three test group-talks included 4, 9 and 12 participants respectively.
  The outline of the study protocol and MI intervention with intended goals of each contact and the time-line is shown in Table 1.

**Table 1:** Summary of the MI intervention / study protocol

Mode of contact, venue & time-line	Objective
First contact *Test-group* - MI group-talk Given at preschools by MI counsellor / educator (RN) and Dental Nurse	For *Test-group* - Establish rapport and discuss oral health goals. Introduce dental health menu items. Use of MI to support change to participant’s oral heath behaviour (OARS approach). Given written dental health information.
*Control-group* - DHE group-talk Given at preschools by Dental Nurse.	For *Control-group*–delivery of verbal and written dental health information.
Oral health questionnaire given to all participants in *Test and Control-group*.	Collection of baseline data on oral health knowledge, attitudes and behaviour & Readiness Assessment of Parents Concerning Infant Dental Decay (RAPIDD)
1^st^ MI follow-up with *Test-group* 2 weeks after MI group-talk. (telephone contact by Dental Nurse)	As part of MI, provide support and encouragement for participants. Re-establish oral health goals and commitment to items selected from oral health menu options. Help to solve problems encountered.
2^nd^ MI follow-up to *Test-group* 1 month after MI group-talk (telephone contact by Dental Nurse) End of MI intervention	As part of MI, promote maintenance of any positive changes made to oral health behaviour. If needed offer further advice and help with problems encountered.
Oral health questionnaires to all participants in *Test and Control-group* (4 months after MI group-talk)	Collection of questionnaire data: Oral health knowledge, attitudes and behaviour. & RAPIDD.
Focus group with a sub-sample of participants in Test-group at preschool. (7 months after MI group-talk)	Collection of qualitative data on participant experience of the MI intervention.

### Details of the MI group-talk

The MI provider established rapport by showing concern and getting the parent/caregiver to talk about their child’s oral health and their goals for their own and their child’s oral health and oral healthcare (using open-ended questions and affirming positive efforts). Questions were themed around the following topics: Eliciting commitment to change, identifying potential problems, enhancing commitment to change, and recognizing resistance to change.

Examples of these questions were:

*Tell me about your child? What do you want for your child’s oral health teeth? “What are your worst fears about your child’s teeth? What are you dental care challenges? How would you like things to have turned out? How could it be better? “What do you want for your child’s teeth in the future?” If you could have one with wish for your child’s teeth, what would it be?”*

• Paraphrasing the parent/caregiver’s wants and desires for their child’s oral health. (Using reflective listening and summarizing parent/caregiver goals). For example:

*“Thank you for telling me about your child, what I understand is that you would like your child to……is that accurate?”*

• Presenting dental health menu options.

Participants in the MI group were shown and encouraged to share their thoughts about a written list of preventive options, termed the Dental Health Menu (Table 2). These options were based on those listed in the Weinstein protocol [18] but modified to take account of participants with older children, along with items based on views expressed by parents and caregivers of preschool children from previous research in Trinidad [17]. From this list, participants were asked to choose and to commit to items they felt able to, as part of the intervention and follow-up.

**Table 2:** The Dental Health Menu (participants could select some or all of the advice options)

• If breastfeeding discontinue.
• Stop bottle feeding (switch to cup).
• Don’t give sugary drinks at night.
• Limit sweet drinks to mealtimes and try to give natural fruit juice instead of colas or other sweet drinks.
• Help to brush your child’s teeth twice a day (one of which should be at bedtime)
• Help to brush from behind your child.
• Use fluoride toothpaste (pea size amount)
• Limit sweet snack to no more than three times a day at mealtime.
• Use fruits and savoury snacks instead of chocolates and candies.
• Register child with a dental clinic.
• Take your child for a dental check-up and fluoride varnish treatment every six months.

### Telephone follow-up

As well as the MI group-talk at baseline, for those included in the test group, there was a follow-up of participants via telephone contact at two weeks and at one month. This was to maintain contact between participants and the MI team, problem solve, reinforce commitment and provide support. Without follow-up, new behaviours may not be tried out or the new behaviour may be tried out but not maintained because of (a) unanticipated problems (b) new behaviour was not integrated into daily routine causing relapse [12]. The telephone follow-up was undertaken by a dental nurse who had been taking field notes.

Two dental nurses were trained for this phase of the intervention by the MI counsellor /educator (RN) in a one-hour, face-to-face training session that included reviewing the written follow-up protocol. This document contained a telephone ‘script’ used in the Weinstein protocol. It was agreed that the script was not to be followed word-for-word but used as a basis / reference point for the telephone conversations.

Questions included in the telephone follow-up included the following: *“I am calling to go over your plan (dental health menu choices).” “Let’s go over parts of the plan that are a problem, sometimes a small adjustment can make a big difference” “Another mother I spoke to, had a similar problem …she tried* (insert example)*…it seemed to work for her family…you are the expert on your family, what do you think?”* [18]

### Instruments

At baseline and 4 months later, parents and caregivers were asked to complete a self-administered oral health questionnaire This instrument contained two main elements (a) Oral health knowledge, attitude and behaviour and (b) An assessment of ‘readiness for change’.

- Oral health knowledge, attitude and behaviour
  The oral health section of the instrument included demographic information, along with a questionnaire on oral health knowledge, beliefs and attitudes, previously used in research among families with young children attending a dental hospital in Trinidad [19]. All questions in this instrument were included in the present study. Additional questions on brushing frequency *(how many times in the last week did your child brush his/her teeth in the last week?),* oral health self-efficacy (OHSE) and oral health fatalism (OHF) were item subscales used in a study of children aged 1–5 from low-income African American children in the USA (Detroit Dental Health Project), which were found to have good internal reliability and validity [20, 21].
  The adapted instrument in the present study (i.e. combination of the Trinidad questionnaire and the question items from the Detroit questionnaire) was reviewed for face validity by the local research coordinators (RN, JN) and found to be appropriate for language and question structure.
  OHSE was scored as follows: *How confident are you that you can get your child’s teeth brushed at bedtime in the following situations?: When under a lot of stress, when feeling low, when feeling anxious, feeling too busy, when feeling tired, worrying about things, when your child doesn’t stay still when you you want to brush them, when told by your child he/she does not feeling brushing.*
  For each of these items the responses were recorded on a 4-point scale: (4 = Very confident to 1 = Not all confident). OHF was measured on a 5-point scale, 5 = Strongly agree to 1 = Strongly disagree, for the following statements: ‘*Most children eventually develop cavities’, ‘Cavities in Baby teeth don’t matter since they fall out anyway’*.

(b) The Readiness Assessment of Parents Concerning Infant Dental Decay (RAPIDD)
  The parent/ caregiver oral health questionnaire also included a specific instrument, the Readiness Assessment of Parents Concerning Infant Dental Decay (RAPIDD) developed by Weinstein and Reidy [22]. Based on the Transtheoretical /stages of change model (TTM), this instrument was designed to measure parent/caregiver ‘*readiness for change’*. Using four constructs: Openness to Health Information, Valuing Dental Health, Convenience and Change Difficulty and Child Permissiveness, RAPIDD attempts to assess whether the parent/caregiver is at one of the following stages: *pre-contemplative, contemplative, preparing for action*, with respect to their child’s oral healthcare. The RAPIDD instrument was validated in a study among families with young children (age 6 to 36 months) in the US Commonwealth of the Northern Mariana Islands, in the Pacific. Again, for use in the present study, the instrument was assessed for face validity and modified for use with a slightly older age-group (3 to 5 years) by the authors (RN, JN). This included rewording of questions relating to bottle-use. Table 3 shows the four RAPIDD constructs and corresponding construct items. It should be noted that, within the questionnaire, these items are not themed by construct but listed as statements for which the participant is asked to agree with on a 5-point Likert scale (5 = Strongly Agree, 4 = Agree, 3 = Neither agree or disagree, 2 = Disagree, 1 = Strongly Disagree). Mean scores are derived for each of the four RAPIDD constructs.

**Table 3:** Modified RAPIDD constructs

Construct	Construct items
Openness to health information	• I would take my child off the bottle if a healthcare professional told me to do so.
	• I get advice on taking care of my baby from TV, radio, magazines and internet.
	• I feel comfortable asking a healthcare professional about ways to care for my child.
	• It is easier for me to get answers about ways to take care of my child from the healthcare professional
Valuing Dental Health	• Keeping my child’s teeth healthy is important to me.
	• My child benefits a lot when I clean his/her teeth.
	• I like the idea of a dentist or dental nurse putting fluoride on my child’s teeth to protect them from cavities
	• I believe using fluoride toothpaste every day would help my child’s teeth
Convenience and Change Difficulty	• It would be hard to give my child less sweets.
	• My child gives me a hard time when I try to brush his/her teeth.
	• It is not easy to use fluoride toothpaste every day.
	• I am unable to put my child to sleep without feeding him/her.
Child Permissiveness	• Food and drinks that are not sweet don’t taste good to my child.
	• I feel like a bad parent if I don’t give my child sweets.
	• My child is happier when I give him/her something sweet in the bottle.
	• I makes me feel good to give my child something sweet to eat.
  In the present study, RAPIDD data were used to assess participant’s ‘readiness for change’ as an outcome measure of the MI intervention based on mean scores for the above-listed constructs and identified as pros and cons. As parents and caregivers weigh both the pros and cons of changing their behaviour, tipping the balance in favour of pros and reducing the cons may facilitate behaviour change. Two constructs assess pros: ‘*Openness to Health Information’* and *‘Valuing Dental Health’* and two constructs assess cons: ‘*Convenience and Change Difficulty’* and *‘Child Permissiveness’.*

### Statistical analysis

The Chi square test was employed for categorical variables related to oral health knowledge (baseline versus follow-up), for test and control-group. These were: *causes of caries, toothbrush size, brushing position, diet / frequency of sweet snacks, toothpaste and fluoride use* (significance level: *p* < 0.05).

Independent sample *t*-test was employed to assess the effect of the intervention on continuous variables related to oral health behaviours and attitudes. These were *Tooth brushing frequency, Oral health Self-efficacy, Oral health fatalism, and RAPIDD constructs*. Mean scores for these items were compared at follow-up between test and control-group (significance level: *p* < 0.05).

### Qualitative study (Focus groups)

The present study used a focus group approach to collect qualitative data on participants’ experiences with the MI intervention. Focus groups are one of several methods for acquiring qualitative data. They can be considered as an semi-structured interview with a group of people who are encouraged to interact with each other and the facilitator, using group dynamics to stimulate discussion, gain insights and generate ideas to explore a chosen topic in depth.

### Focus group sample selection

In liaison with the preschool head teachers / administrators, a focus group discussion was arranged for parents/caregivers from one of the three preschool (clusters) that had received the MI intervention. This took place 6 months after the end of MI intervention (i.e. last telephone follow-up). Due to the busy schedule of events in the other two preschools their head-teachers /administrators indicated that they were unavailable for inclusion in this phase of the study. All parents /caregivers from the preschool who had attended the group-talk and engaged in the follow-up were invited to participate, through the head-teacher/ administrator. The venue for the focus group discussion was the preschool main classroom, after working hours, during the middle of a school week.

The focus group was run by a facilitator / moderator (RN), previously trained in qualitative methodology (26 h, didactic and practical,) and a dental nurse, who served as the assistant moderator.

### Conduct of the focus group

The facilitator/ moderator, welcomed the parent’s and caregivers to the event. To obtain informed consent for participation in the focus group, the purpose and conduct of the event (focus group) was explained i.e. an open discussion (not an interview) around the theme of preschool children’s dental healthcare based on the dental health group-talk and telephone calls given some months previously. It was explained that an audio recording of the discussion would be made but that all comments and opinions offered would remain anonymous and individuals would not be identified by name during transcription and reporting of the findings. It was also emphasized that all comments (positive or negative) would be considered of value. All the parents and caregivers present gave verbal consent to participate in the focus group discussion. Participants were also invited to complete a short form to record socio-demographic information.

To initiate and facilitate the discussion the moderator used a *focus group topic guide* (Table 4). This topic guide was designed to help explore how participants felt about receiving the MI intervention (group talk, dental health menu and telephone follow-up), along with specific issues related to making changes to oral healthcare practices and routines following the intervention. Questions in the topic guide were open-ended and not asked in a specific order but rather in response to the flow of the discussion.

**Table 4:** Focus group topic guide

• After the group talk how confident were you in making any changes?
• Did any of the things we talked about help you to make a change? (If yes, what were they/)
• What did you find most difficult to change?
• What were the easiest things to change?
• What experiences did you have with tooth brushing or changing the type of feeding?
• Did any of you have to change from a feeding bottle to a cup? How was that for you?
• Did the talk feel different to any dental talks / advice you had before? (If so how?)
• Was the dental health menu helpful? (If so how?)
• What did you feel about the telephone follow-up calls (Were they helpful)?
• Overall how have you been managing with your child’s oral healthcare and are there any problems you are still having?

The discussion was recorded on a digital audio recorder and field notes taken by the assistant moderator. At the end of the session, participants were thanked and given dental products as tokens of appreciation (toothpaste / floss samples). Light refreshments were provided before and during the discussion.

### Qualitative data analysis

A thematic content analysis was used to analyse the qualitative data collected in the focus group session. As a first step, the focus group audio recording was transcribed into a Word document. The verbatim transcript was then numbered line-by-line for identification. After several readings of the transcript, field notes and listening to the audio recording, the data underwent a process of initial labelling of sections of the transcript by placing codes in the text margin. Similarly coded pieces of the transcript were re-assembled near to each other by cutting and pasting text sections into a new Word document. These initial codes (which could overlap) were then further developed to identify emerging early ‘themes’ (proto-themes) which were themselves further refined into the final themes These themes were reported in the results with supporting verbatim quotes from the transcript.

### Data trustworthiness and reflexivity

To determine data credibility (member checking), at the end of the session participants were invited to give their opinion (off-record) as to whether the discussion had accurately documented their own experiences [23]. All focus group participants agreed that the points discussed and views presented had been a good representation of their collective and individual experiences with the MI intervention. Reflexivity in qualitative research recognises that the researcher is part of the process of producing the data and interpreting their meaning [23, 24]. Furthermore, consideration was given to the facilitator /moderator having also been an active part of the MI intervention. This was not however, believed to have influenced the views expressed in the focus group discussion or subsequent interpretation of the data.

## Results

The following section describes the results of the MI intervention study, from the data gathered in the oral health questionnaire as well as the results for the qualitative study, based on a thematic analyses of the focus group data.

### Intervention study enrolment and retention

From 150 parents/caregivers that were invited, 79 agreed to participate in the study (response rate 53 %). From this initial study enrolment, at follow-up 20 and 21 participants remained in the test and control-groups, respectively (Fig. 1). A significantly higher proportion of participants dropped out of the control-group compared to the test-group by the end of the study (Chi Square *p* < 0.001). Based on post-hoc analysis the power of this study was 61 %.

### Sample characteristics

Table 5 shows the sample characteristics at baseline. The majority of the participants were in the 25–34 year age range indicating a sample of young adults as would be expected among parents of preschool age children. The majority were females of mixed ethnicity and most participants were in manual employment or housewives. Most participants were educated to secondary level and cared for 1–2 children. At baseline in the test-group there were significantly less participants of African ethnicity, a greater proportion employed in professional / managerial jobs and having tertiary level education, compared to the control-group (Chi square *p* < 0.05). Compared to the control group, a significantly greater proportion of participants in the test-group did not have a regular dental clinic for their child and reported finding it easier to find a medical doctor than a dentist (Chi square *p* < 0.05)

**Table 5:** Sample characteristics at baseline

	Control		Test		All	
Age group	n	%	n	%	n	%
18-24	9	16.7	1	4.0	10	12.7
25-34	22	40.7	16	64.0	38	48.1
35-44	17	31.5	5	20.0	22	27.8
45-54	2	3.7	2	8.0	4	5.1
55-64	2	3.7	0	0.0	2	2.5
0ver 65	1	1.9	0	0.0	1	1.3
Missing	1	1.9	1	4.0	2	2.5
Total	54	100	25	100	79	100
Not significant (Chi square)						
Gender						
Female	45	83.3	21.0	84.0	66	83.5
Male	7	13.0	13.0	16.0	11	13.9
Missing	2	3.7	3.7	0.0	2	2.5
Total	54	100	100	100	79	100
Not significant (Chi square)						
Ethnic group						
African	24	44.4	7	28.0	31	39.2
Indian	3	5.6	7	28.0	10	12.7
Mixed	26	48.1	9	36.0	35	44.3
Missing	1	1.9	2	8.0	3	3.8
Total	54	100	25	100	79	100
*p* < 0.05 (Chi square)						
Occupational group						
Professional	1	1.9	2	8.0	3	3.8
Managerial / lower professional	6	11.1	9	36.0	15	19.0
Non manual (skilled)	13	24.1	3	12.0	16	20.3
Skilled manual	6	11.1	2	8.0	8	10.1
Semi-skilled manual	3	5.6	1	4.0	4	5.1
Non-skilled manual	7	13.0	0	0.0	7	8.9
Housewife/ unemployed	13	24.1	7	28.0	20	25.3
Missing	5	9.3	1	4.0	6	7.6
Total	54	100	25	100	79	100
*p* < 0.05 (Chi square)						
Highest level of education						
University	8	14.8	9	36.0	17	21.5
Technical college	6	11.1	4	16.0	10	12.7
Secondary	32	59.3	9	36.0	41	51.9
Primary	7	13.0	2	8.0	9	11.4
Missing	1	1.9	1	4.0	2	2.5
Total	54	100	25	100	79	100
*p* < 0.05 (Chi square)						

### Oral health knowledge

Table 6 describes the effect of the MI and traditional DHE, on oral health knowledge in the test and control-groups respectively, at 4-month follow-up. Compared to baseline, in the control-group there was a significant increase in the proportion of participants who correctly knew that their child’s teeth should be brushed from behind and that fluoride varnish should be administered every six months.

**Table 6:** Oral health knowledge for control group and test-group – baseline versus follow-up (Chi square test)

Question		Control Baseline		Control Follow-up		*p* value	Test Baseline		Test Follow-up		*p value*
		n	%	n	%		n	%	n	%	
Bacteria on the teeth of young children can cause cavities	Yes	50	92.6	18	90.0		22	88.0	18	85.7	
	No	1	1.5	1	5.0	0.758	0	0	0	0	0.819
	Don’t know	3	5.6	1	5.0		3	12.0	3	14.3	
What size of toothbrush is best for a young child	Small	42	79.2	18	90.0		19	79.0	17	81.0	
	Medium	8	15.1	2	10.0	0.447	4	16.7	4	19.0	0.633
	Don’t know	3	5.7	0	0		1	5.3	0	0	
How much toothpaste should be placed on the brush	Enough to cover brush	10	18.5	3	15		6	24	0	0	
	Pea size	33	61.1	17	85	0 .144	14	56.0	19	90.5	
	Smear	8	14.8	0	0		2	8.0	2	9.5	<0.05
	Don’t know	3	5.6	0	0		3	12.0	0	0	
From what position should you help to brush	In front of the child	35	64.8	9	45.0		11	44.0	0	0	
	Behind the child	9	16.7	9	45.0		6	24.0	19	90.5	<0.001
	From the side	4	7.4	2	10.0	<0.05	4	16.0	2	9.5	
	Don’t know	6	11.4	0	0		4	16.0	0	0	
How much fluoride should be in the toothpaste	Not less than 1000 ppm	1	1.9	1	5.0		1	4.0	1	4.8	
	450-600 ppm	3	5.6	2	10.0	0.590	0	0	4	19.0	0.071
	Don’t know	50	92.6	17	85.0		20	83.3	16	76.2	
Fluoride varnish should be placed on teeth every 6 months	Yes	2	3.7	10	50.0		4	16.7	16	76.2	
	No	8	14.8	1	5.0	<0.05	0	0	1	4.8	<.0001
	Don’t know	14	81.5	9	45.0		20	83.3	4	19.0	
When is it safe to give sugary drinks and snacks	Between meals	25	46.3	9	45.0		7	28.0	9	42.9	
	At mealtimes	5	9.3	1	5.0		4	16.0	11	52.4	
	At night	0	0	2	10.0	0.200	0	0	0	0	<0.05
	in the morning	7	13.0	3	15.0		3	12	0	0	
	Don’t know	17	31.5	5	25.0		11	44.0	1	4.8	
When should you take your child for their first dental visit	Only if problems	1	1.9	1	5.0		2	8.0	1	4.8	
	By 1 year-old	21	38.9	4	20.0		5	20.0	7	33.2	
	When some adult tooth	1	1.9	1	5.0	0.412	1	4.0	0	0	0.178
	When all baby teeth	20	37.0	11	55.0		8	32.0	11	52.4	
	Don’t know	11	20.4	3	15.0		9	36.0	2	9.4	

In the test-group, compared to baseline, significantly greater proportions of participants, as compared with the test group, knew the correct quantity of toothpaste to use (pea size amount), the correct brushing position, importance of six monthly fluoride varnish application and that main mealtimes were the safest time to give sugary snacks was at mealtimes (Chi square test).

### Oral health behaviour and attitudes and RAPIDD

Table 7 describes the effect of the MI and traditional DHE, on oral behaviours and attitudes at 4-month follow-up. In the test-group there was a significant increase in the child weekly brushing frequency and a significant reduction in oral health fatalism, compared to the control-group (independent sample *t*-test). There were no statistically significant changes in mean scores for the oral health self-efficacy or the RAPIDD constructs / RAPIDD pros and cons.

**Table 7:** Oral health behavior and attitudes at 4-month follow-up, control v test group (Independent sample *t*-test)

Variable	Group	n	Mean	sd	95 %CI	*p* value
Child weekly tooth brushing						
	Control	20	10.55	4.07	8.77-12.33	<0.01
	Test	21	13.09	1.44	12.47-13.71	
Self-efficacy						
	Control	20	24.60	6.91	21.57-27.62	0.379
	Test	21	26.79	5.14	24.59-28.99	
Oral health fatalism						
	Control	20	5.95	2.04	5.06-6.84	<0.05
	Test	21	4.09	1.73	3.35-4.83	
Openness to health information						
	Control	20	15.35	2.64	14.19-16.51	0.593
	Test	21	15.86	3.32	14.73-16.99	
Valuing dental health						
	Control	20	19.80	8.71	16.07-23.52	0.847
	Test	21	19.42	0.81	19.07-19.77	
Convenience and change difficulty						
	Control	19	9.58	2.34	9.23-9.94	0.410
	Test	21	7.67	3.21	6.30-9.04	
Child permissiveness						
	Control	20	8.30	3.86	6.61-9.99	0.352
	Test	21	7.33	3.63	5.78-8.88	
RAPIDD pros						
	Control	20	35.15	9.58	30.95-39.35	0.952
	Test	21	35.29	3.38	33.84-36.74	
RAPIDD cons						
	Control	19	17.37	4.98	15.13-19.61	0.154
	Test	21	15.00	5.28	12.74-17.26	

### Qualitative findings

The following section describes the results of the qualitative study, based on the thematic analysis of the focus group discussion. Six participants from the 20 parents and caregivers who had remained in the MI intervention study at the four-month follow-up, accepted an invitation to take part in the focus group discussion. All participants were female, ranging in age from 25–34, with two thirds (4) of Indian ethnicity. Four participants stated secondary school was their highest level of education the remainder educated to tertiary level. Three participants were in professional / managerial occupation, the others being in manual occupation and housewives. Two participants cared for more than one child.

### Views on the MI group-talk

During the discussion it became apparent that participants had not had any kind of dental health education (DHE) delivered to them prior to the MI group talk, therefore they could not compare it to other types of DHE delivery. However, they all appeared to feel comfortable with the MI approach in particular, the open discussion of oral health goals and barriers along with sharing of experiences with other parents / caregivers. Opinions offered on the telephone follow-up element of the MI protocol suggested that it did not feel intrusive and was helpful; no participants indicated discomfort with that aspect of the MI.

*I think it* (MI group talk) *was better because sometime you might even forget about something to mention and another parent might bring it up and you know you would benefit from it.*

*Yes, it was really good* (MI group talk)*, because I never knew about the fluoride varnish before and I did enquire from my dentist*

*That helped me amm… a lot* (telephone follow-up)*..and making sure that she brushes her teeth twice-a-day… because sometimes you know, they small so you’ll say well if they fall asleep, so we now try to kind of wake her up and brush it…*

### Mode of delivery of oral health information

Mixed opinions were expressed about the value of giving written material but most found written information helpful as a reinforcement / reminder of issues presented in the MI group talk. It was unclear at times if participants were referring to the value of the dental health information leaflet or the dental health menu used as part of the MI, as to some participants they may have seemed similar in content.

*I don’t think it would matter to me to much because I mean at the end of the day we could verify the information* (information leaflet) *on the internet ‘cause everything is there on the internet.*

*I find the talk does be better because sometimes you get the hand-out* (Information leaflet) *and you misplace it*

*You should give both* (MI group talk and written information)

### Changing oral health practices

As a result of the MI intervention most participants had tried to implement some changes to oral health practices for their child such as twice daily tooth brushing, change of brushing position, use of fluoride toothpaste etc. In particular, after the MI intervention some participants appeared to have made concerted effort to overcome practical difficulties such as their child falling asleep before night-time brushing. Following the intervention there also appeared to be greater understanding of the importance of using fluoride toothpaste and supervision of tooth brushing. Despite it being a little challenging, after the intervention, participants seemed to feel more confident in adopting the brushing from behind method. For some participants the follow-up phase of the intervention was particularly helpful to support changes that had been made.

*I did learn from the talk because the amount of toothpaste.. so I changed that…*

*she kind of like wants to brush her teeth on her own now but I still make sure and do with her it at least once-a-day especially by bedtime to make sure and do it properly.*

*How I position the child, ‘cause I used to do it from in front and now I know how to do it from behind so I learnt about that. I guess he is more comfortable with me doing it that way.*

*For me it wasn’t a must before (*night-time brushing*)… then I tried to make sure …*

### Changing dietary practices

Some of the participants had children who were still using the feeding bottle (mainly at night). Though they expressed difficulty changing this behaviour in the past, following the MI intervention new efforts were made to discontinue this practice.

*She still begs for the bottle. Well I threw away the nipple (feeding bottle) recently because it was like really bad but only like in the night.., like if she get up around three o’clock in the morning but we cut it down like real plenty.*

*… but you know when she sleeping and start crying for this bottle, it real hard to go back to sleep. But now I throw away the bottle.*

*She would drink the Milo (cocoa drink) in the bottle but when you give it to her in the pack she would have two or three sips and that is it, she want that bottle..*

### Changing dental attendance behaviour

After the MI intervention some participants had made attempts at taking their child for a dental visit but had been hampered by difficulty in getting appointments. Overall, participants’ awareness of dental service availability for young children appeared to have improved with more considering the dental hospital along with private practice. There was a persisting view that dental attendance was only for problems and, in one instance, an inquiry about fluoride varnish at a private dental practice was met with the general dental practitioner (GDP) not being aware of this as a preventive option for primary teeth.

*Well we brought her to a private dentist and sometimes you have a waiting period, like if it’s a emergency probably have to wait for an appointment.*

*…sometimes you might have to get like four Saturdays after, or something like that….even if the child in pain.*

### Barriers to change

For some participants, the main barrier to changing oral healthcare practices was the child’s non-acceptance of new brushing regimes, toothpaste use and change from feeding bottle to cup. Participants recounted concerted attempts to implement new oral health practices. Sometimes these efforts were further hampered by other family member involvement, in particular grandparents.

*…she not even giving up the comforter right now, she want that to sleep whole night, if that out the mouth, she not sleeping, she will be searching for it on the bed.*

*She just cried and cried…I hide the bottle and give her the cup.., she says “No Mummy I don’t want it”.*

*…she likes to lie down and drink her tea…not sit down. The only way she drinking from a cup is with Milo* (sweetened cocoa drink)*….*

*It does be harder for you too, cause they think they right* (grandparent*s*)*.. I tell them she is three, she supposed to cut off the bottle….*

## Discussion

Although not directly linked to behaviour change, improving the knowledge and awareness of parents and caregivers is a key element of dental prevention in preschool children [25–27].

In this study, participants who received MI showed improved knowledge across a wider range of knowledge items (correct amount of toothpaste, supervised brushing position, fluoride varnish, and safest time to give sugary foods and drinks) compared to those who received traditional DHE. Importantly knowledge of the importance of fluoride varnish application also has practical implications for preventive strategies as this clinical intervention is effective in reducing ECC separately and in combination with fluoride toothpaste [28, 29]. The reduction in prevalence of early childhood caries (ECC) following an MI intervention reported in Asian families in Canada, was largely attributed to a greater uptake of fluoride varnish by those families [13] possibly due to the parents attaching greater value to professional preventive care.

On the issue of tooth brushing practices, understanding these parents and caregivers’ perspective was vital and this aspect formed part of the MI conversation that uses ‘reflective listening’, where, over the course of the session, the educator / counsellor seeks to increase the clients strength for expressed motivation in order to target a behaviour change. Establishing good tooth brushing habits and routines in preschool children can be a challenge for families particularly where it involved changes to established daily routines [30].

Regular (at least twice daily) brushing with fluoride toothpaste is a core aspect of caries prevention in preschool children [31, 32, 27]. Importantly, in this present study, the MI intervention had a significant effect on this preventive oral health behaviour, with the test-group claiming an increased frequency of weekly brushing. Brushing with fluoride toothpaste before the child’s evening bedtime gives further protection, as during this time the teeth are more vulnerable to the effects of plaque bacteria [26]. Also, from the views expressed in the focus group it could be inferred that after the intervention, participants felt more confident in their ability to use the oral health information that was provided. Exploring practical ways to overcome barriers to oral healthcare for their child/children may also have helped participants to persist with introducing new oral healthcare regimes about which they had learned. However, getting the child to brush at night-time was one of the more difficult behaviours for parents and caregiver to change, often because the child refused or fell asleep. This is consistent with findings from the UK, where parents of preschool children found more difficulty in establishing a routine of tooth brushing at that time of day [30]. Again, based on findings from the focus group, it appeared that some participants in the test-group had experienced success with efforts to stop use of the night-time feeding bottle. This was encouraging as this routine may also have been a particularly difficult one to change as bottle-use has been associated with issues of closeness and comforting by the mother, as well as ‘buying time’ time away from their crying child [33].

Miller and Rollnick state that “for a person to change they must feel both confident in their ability to change and believe the change is important to them” [34]. Brushing behaviour has been reported to be positively related to maternal self-efficacy [21], therefore, increasing parental involvement in tooth brushing for preschool children would requires them knowing it is important [35]. In the present study, differences in self-efficacy did not reach statistical significance at the four-month follow-up. However, an MI approach significantly reduced parent/caregiver OHF which in turn, may lead to a more positive attitudes toward oral health and increased motivation to adopt preventive practices [15]. Therefore, how BCTs are delivered may have as much impact on outcomes as the technique itself, suggesting the need for development of a BCT Taxonomy (i.e. a method of specifying, evaluating and implementing behaviour change interventions) [6].

The concept of ‘readiness for change’, in this study was explored using the RAPIDD instrument. ‘Readiness for change’ has been defined as ‘a person’s current thoughts, feelings and attitudes regarding their intention to institute a change in their habits’ [34]. The analyses did not demonstrate a statistically significant effect of MI on the RAPIDD construct scores. The present findings are also similar to those of Freudenthal who reported an effect of improvement in brushing frequency in mothers who received MI but, apart from ‘valuing dental health’, reported no significant changes in the RAPIDD scores between test and control-groups [36].

A systematic review, of parental influences on ECC, highlighted the importance of socio-cultural factors that are likely to affect mediating variables such as attitudes and oral health beliefs [37] and it has also been suggested that the MI approach enables oral health professionals to focus more on underlying social determinants of disease, by tailoring interventions to suit social circumstances [38] while attempting to develop their ‘readiness for change’.

From another perspective, it has been suggested that MI provides an opportunity to address ‘felt needs’ [39], which supports a view that primary healthcare professionals should address the needs of families in the context of their environment and experience [40]. It has also been suggested that health practitioners are likely to gain a greater sense of achievement from recognising progress in an individual’s ‘readiness for change’ as an important outcome rather than using behaviour change as the only goal since this may not be measurable in the short term [41].

The MI intervention led to a much higher retention rate of participants in the study, compared to the control-group. This may be a benefit of the MI approach. Importantly, comments from the focus group indicated participants in the test-group were comfortable with the telephone contacts made by the Dental Nurses suggesting that this element of the protocol may have helped to maintain motivation and commitment from participants. This is consistent with the findings of Weinstein, who believed that longer telephone or in-person follow-up could add even more value to the process [11]. Also the challenges the participants in the present study encountered, in trying to alter dietary and dental attendance behaviour, indicates the value of on-going support to achieve these goals.

### Limitations of the study

Some limitations of the present study must be considered when interpreting the findings.

1. Sample sizes and subsequent group allocations were relatively small due to difficulty in subject recruitment and retention, which has implications for potential coverage of this type of community intervention. The low response rate may have also have resulted in a more motivated group of participants at baseline compared to the target population, masking effects of the intervention. Furthermore, the sample size in this study resulted in a low study power increasing the probability of type II errors.
2. In the present study lack of a clear effect of MI on participants on oral health attitudes and behaviours may also have been due to the extent the actual MI delivered matched the principles of MI i.e. fidelity . Fidelity refers to assessment and monitoring of an intervention as it is actually delivered to clients and its assessment is key to the validity of behavioural change interventions [42]. Interestingly, one of the reasons suggested for lack of effectiveness in improving oral health in one study of preschool children was lack of fidelity to the MI process [14]. With regards to this, Moyers et al. have developed an instrument to help clinicians improve in their use of MI by providing feedback by use of the Motivational Interviewing Treatment Integrity (MITI) code [43]. For the MITI, recordings of the MI session are reviewed by trained coders who assign a global score based on a 5-point scale for an overall impression and for each of five specific dimensions: Evocation, Collaboration, Autonomy/ Support, Direction and Empathy [43].
3. Differences in characteristics between the test and control-group at baseline (education level, occupation, experiences of accessing dental care) may have influenced the effect of the intervention. Although the groups were randomly allocated, these socio-demographics imbalances may have been due to some preschools having significantly more parents from a particular ethnic or socioeconomic group. This also prevents more sophisticated analyses.
4. The duration of the study was short (4 months). This was to avoid further drop-out and loss of contact with participants as their children moved on to primary school / families leave the area.
5. Self-reported questionnaires can be affected by participant recall [44]. Furthermore, some response bias may have resulted from ‘social desirability’ i.e. bias due to clients who misrepresent self-reported behaviours by over-reporting behaviours considered socially desirable, and under-reporting undesirable ones [45].
6. As only one individual was trained to provide the MI talk, the MI method may not be representative of other MI providers, which limits the ability to generalize conclusions about its utility.
7. More extensive training in MI for the dental nurses who undertook the telephone follow-up may have helped increase fidelity to the MI protocol.
8. Delivery of MI in group settings has not been as extensively evaluated as one-to-one counselling, therefore MI delivered in a group setting has less predictable outcomes [7]. Specific limitations being: (a) each group member has less time to engage in the process of evoking and change-talk (b) Group dynamics can alter the probability of change-talk and (c) people within the group may reinforce ambivalence in others.
9. It was not possible to include all parents and caregivers who received the MI in the focus group discussion as most were unavailable. This limited the sample size and group size of the focus group. However, minimum sample size for focus group has been stated as six [45] and, where there is a high degree of homogeneity for qualitative studies, samples of less than ten can still achieve data saturation [46]. Also, as a focus group was not conducted with the control-group it cannot be concluded whether participants' experiences of MI were more positive in comparison to traditional DHE.
10. The impact of the MI intervention compared to the control group may have been exaggerated as DHE is known to be ineffective for changing oral health behaviour.

## Conclusion

Although the findings should be considered as exploratory, in this controlled study there was some evidence that using an MI approach when delivering oral health information had a positive effect on parent/ caregiver oral health knowledge, tooth brushing behaviour and oral health fatalism compared to traditional DHE. The acceptability of the MI protocol, which included follow-up telephone contact was also good, as indicated by discussions with the recipients both during and after the delivery of the programme. These findings suggest that further development of this person-centred counselling approach could be of benefit for improving oral health of preschool children in Trinidad and could form part of oral health promotion strategies for this population group.

## Abbreviations

BCT: Behaviour change technique
DHE: Dental health education
ECC: Early childhood caries
GDP: General dental practitioner
MI: Motivational interviewing
OHF: Oral health fatalism
OHSE: Oral health self-efficacy
RAPIDD: Readiness assessment of parents concerning infant dental decay
TTM: Transtheoretical model.
